# The association between iron deficiency and outcomes: a secondary analysis of the intravenous iron therapy to treat iron deficiency anaemia in patients undergoing major abdominal surgery (PREVENTT) trial

**DOI:** 10.1111/anae.15926

**Published:** 2022-12-08

**Authors:** T. Richards, L. F. Miles, B. Clevenger, A. Keegan, S. Abeysiri, R. Rao Baikady, M. W. Besser, J. P. Browne, A. A. Klein, I. C. Macdougall, G. J. Murphy, S. D. Anker, D. Dahly, The PREVENTT trial collaborators, Toby Richards, Martin Besser, John Browne, Ben Clevenger, Anastazia Kegan, Andrew Klein, Lachlan Miles, Iain MacDougall, Ravishankar Rao Baikady, Darren Dahly, Andrew Bradbury, Toby Richards, Trevor Burley, Shelley Van Loen, Stefan Anker, Andrew Klein, Iain MacDougall, Gavin Murphy, Martin Besser, Isabel Unsworth, Tim Clayton, Tim Collier, Kimberley Potter, Sandy Abeysiri, Richard Evans, Rosemary Knight, Rebecca Swinson, Laura Van Dyck, Jane Keidan, Lorna Williamson, Angela Crook, John Pepper, Joanna Dobson, Simon Newsome, Tom Godec, Matthew Dodd, Toby Richards, Laura Van Dyck, Richard Evans, Sandy Abeysiri, Ben Clevenger, Anna Butcher, Rebecca Swinson, Tim Collier, Kimberley Potter, Stefan Anker, John Kelly, Steven Morris, John Browne, Jane Keidan, Michael Grocott, Marisa Chau, Rosemary Knight, Timothy Collier, Ravishankar Rao Baikady, Ethel Black, Helen Lawrence, Maria Kouthra, Katherine Horner, Sham Jhanji, Ed Todman, Zoe Keon‐Cohen, Martin Rooms, Judith Tomlinson, Ian Bailes, Susanna Walker, Katrina Pirie, Michelle Gerstman, Ramanathan Kasivisvanathan, Sophie Uren, David Magee, Alex Eeles, Rob Anker, Jamie McCanny, Michelle O'Mahony, Toby Reynolds, Sian Batley, Aoife Hegarty, Simon Trundle, Francesca Mazzola, Kate Tatham, Alina Balint, Ben Morrison, Matthew Evans, Ching Ling Pang, Lorna Smith, Charlotte Wilson, Victoria Sjorin, Poonam Khatri, Marco Wilson, David Parkinson, James Crosbie, Khaled Dawas, Deborah Smyth, Georgia Bercades, Jung Ryu, Anna Reyes, Gladys Martir, Laura Gallego, Alison Macklin, Magda Rocha, Dr Karen Tam, Dr David Brealey, Jugdeep Dhesi, Catriona Morrison, Joanna Hardwick, Jude Partridge, Philip Braude, Andrew Rogerson, Nymah Jahangir, Clare Thomson, Lizzie Biswell, Jason Cross, Ffion Pritchard, Aminata Mohammed, Deirdre Wallace, Ma Gem Galat, Jane Okello, Rebecca Symes, Josette Leon, Charlotte Gibbs, Sumayer Sanghera, Andy Dennis, Faith Kibutu, Joyce Fofie, Sarah Bird, Abiola Alli, Yvonne Jackson, Salah Albuheissi, Carol Brain, Connie Shiridzinomwa, Catherine Ralph, Belinda Wroath, Fiona Hammonds, Benita Adams, John Faulds, Sara Staddon, Timothy Hughes, Sian Saha, Clare Finney, Clair Harris, Clare Mellis, Lucy Johnson, Paul Riozzi, Adam Yarnold, Fraser Buchanan, Philip Hopkins, Louise Greig, Harriet Noble, Mark Edwards, Mike Grocott, James Plumb, David Harvie, Ahilanandan Dushianthan, Mai Wakatsuki, Samantha Leggett, Karen Salmon, Clare Bolger, Rachel Burnish, James Otto, Gurinder Rayat, Kim Golder, Pauline Bartlett, Sitara Bali, Leanne Seaward, Beverley Wadams, Bryony Tyrell, Hannah Collins, Natasha Tantony, Rosie Geale, Amber Wilson, Darran Ball, Ian Lindsey, Debbie Barker, Madeleine Thyseen, Patrick Chiam, Carol Hannaway, Kerry Colling, Cheryl Messer, Neil Verma, Mariam Nasseri, Gail Poonawala, Abbie Sellars, Pratyuja Mainali, Toby Hammond, Al Hughes, David O'Hara, Fiona McNeela, Lauren Shillito, Alwyn Kotze, Catherine Moriarty, Jonathan Wilson, Simon Davies, David Yates, Joe Carter, Jon Redman, Sara Ma, Kate Howard, Heidi Redfearn, Danielle Wilcock, Justine Lowe, Tamara Alexander, Jasmine Jose, Gillian Hornzee, Fatima Akbar, Severine Rey, Anoop Patel, Samantha Coulson, Rajan Saini, Joseph Santipillai, Thomas McCretton, Jamie McCanny, Kiran Chima, Karen Collins, Byiravey Pathmanathan, Anjalee Chattersingh, Laura McLeavy, Zayneb Al‐Saadi, Manju Patel, Sofia Skampardoni, Rajkumar Chinnadurai, Vicky Thomas, Anne Keen, Katherine Pagett, Clare Keatley, Jason Howard, Marie Greenhalgh, Stephen Jenkins, Ranjit Gidda, Angela Watts, Chris Breaton, Jane Parker, Susan Mallett, Sarah James, Lisa Penny, Kim Chan, Tamsin Reeves, Marisa Catterall, Sue Williams, Janine Birch, Kate Hammerton, Nicola Williamson, Anni Thomas, Melanie Evans, Lily Mercer, Gill Horsfield, Claire Hughes, Jason Cupitt, Emma Stoddard, Helen McNamara, Chloe Birt, Alexander Hardy, Robert Dennis, Deborah Butcher, Susie O'Sullivan, Alan Pope, Sumaya Elhanash, Stephen Preston, Helen Officer, Andrea Stoker, Stuart Moss, Alison Walker, Anna Gipson, Julie Melville, Joanne Bradley‐Potts, Richard McCormac, Vivienne Benson, Kirsty Melia, Julie Fielding, Wendy Guest, Simon Ford, Henry Murdoch, Susan Beames, Paula Townshend, Kayleigh Collins, Jon Glass, Bethan Cartwright, Balsam Altemimi, Lucy Berresford, Chris Jones, Leigh Kelliher, Sam de Silva, Katie Blightman, Kate Pendry, Lebina Pinto, Shubha Allard, Louise Taylor, Ahmed Chishti, Julia Scott, Debbie O'Hare, Michael Lewis, Zahid Hussain, Karen Hallett, Susan Dermody, Carolyn Corbett, Louise Morby, Matthew Hough, Sarah Williams, Patricia Williams, Sarah Horton, Pauline Ashcroft, Anthony Homer, Alastair Lang, Heidi Dawson, Ewen Harrison, John Thompson, Vimal Hariharan, Vanessa Goss, Ramachandran Ravi, Georgina Butt, Mark Vertue, Austin Acheson, Oliver Ng, Debbie Bush, Edward Dickson, Amy Ward, Sophie Morris, Andrew Taylor, Rebecca Casey, Lawrence Wilson, Dale Vimalachandran, Maria Faulkner, Helen Jeffrey, Claire Gabrielle, Sharon Martin, Andrew Bracewell, Jenny Ritzema, David Sproates, Farhad Alexander‐Sefre, Christiane Kubitzek, Sally Humphreys, James Curtis, Paul Oats, Sandra Swann, Abbie Holden, Claire Adam, Louise Flintoff, Claudia Paoloni, Karen Bobruk

**Affiliations:** ^1^ Division of Surgery University of Western Australia, Perkins South Building, Fiona Stanley Hospital, Murdoch Perth WA Australia; ^2^ Institute of Clinical Trials and Methodology and Division of Surgery University College London UK; ^3^ Department of Critical Care Melbourne Medical School, The University of Melbourne VIC Australia; ^4^ Department of Anaesthesia Austin Health Melbourne VIC Australia; ^5^ Department of Anaesthesia Royal National Orthopaedic Hospital Stanmore UK; ^6^ Department of Haematology, PathWest Laboratory Medicine King Edward Memorial Hospital Subiaco WA Australia; ^7^ Division of Surgery University of Western Australia, Perkins South Building, Fiona Stanley Hospital, Murdoch Perth WA Australia; ^8^ Department of Anaesthesia The Royal Marsden NHS Foundation Trust London UK; ^9^ Department of Haematology Addenbrooke's Hospital Cambridge UK; ^10^ School of Public Health University College Cork Ireland; ^11^ Department of Anaesthesia and Intensive Care Royal Papworth Hospital Cambridge UK; ^12^ Department of Renal Medicine King's College Hospital London UK; ^13^ Department of Cardiovascular Sciences University of Leicester UK; ^14^ Department of Cardiology Berlin Institute of Health Centre for Regenerative Therapies; German Centre for Cardiovascular Research partner site Berlin; Charité Universitätsmedizin Berlin Germany; ^15^ Health Research Board Clinical Research Facility University College Cork Ireland

**Keywords:** anaemia, iron, transfusion

## Abstract

In the intravenous iron therapy to treat iron deficiency anaemia in patients undergoing major abdominal surgery (PREVENTT) trial, the use of intravenous iron did not reduce the need for blood transfusion or reduce patient complications or length of hospital stay. As part of the trial protocol, serum was collected at randomisation and on the day of surgery. These samples were analysed in a central laboratory for markers of iron deficiency. We performed a secondary analysis to explore the potential interactions between pre‐operative markers of iron deficiency and intervention status on the trial outcome measures. Absolute iron deficiency was defined as ferritin <30 μg.l^−1^; functional iron deficiency as ferritin 30–100 μg.l^−1^ or transferrin saturation < 20%; and the remainder as non‐iron deficient. Interactions were estimated using generalised linear models that included different subgroup indicators of baseline iron status. Co‐primary endpoints were blood transfusion or death and number of blood transfusions, from randomisation to 30 days postoperatively. Secondary endpoints included peri‐operative change in haemoglobin, postoperative complications and length of hospital stay. Most patients had iron deficiency (369/452 [82%]) at randomisation; one‐third had absolute iron deficiency (144/452 [32%]) and half had functional iron deficiency (225/452 [50%]). The change in pre‐operative haemoglobin with intravenous iron compared with placebo was greatest in patients with absolute iron deficiency, mean difference 8.9 g.l^−1^, 95%CI 5.3–12.5; moderate in functional iron deficiency, mean difference 2.8 g.l^−1^, 95%CI −0.1 to 5.7; and with little change seen in those patients who were non‐iron deficient. Subgroup analyses did not suggest that intravenous iron compared with placebo reduced the likelihood of death or blood transfusion at 30 days differentially across subgroups according to baseline ferritin (p = 0.33 for interaction), transferrin saturation (p = 0.13) or in combination (p = 0.45), or for the number of blood transfusions (p = 0.06, 0.29, and 0.39, respectively). There was no beneficial effect of the use of intravenous iron compared with placebo, regardless of the metrics to diagnose iron deficiency, on postoperative complications or length of hospital stay.

## Introduction

Pre‐operative anaemia is common in patients undergoing non‐cardiac surgery and associated with an increased risk of blood transfusion, hospital length of stay and postoperative complications [1, 2]. The most common cause of pre‐operative anaemia is iron deficiency, which can be caused by reduced or impaired dietary iron absorption, chronic blood loss, or disruption of normal iron metabolism due to comorbidities or inflammation [3]. These aetiologies are commonly seen in patients undergoing major abdominal surgery. Intravenous iron is an effective treatment to increase haemoglobin concentrations [4] and, therefore, a plausible therapeutic option for pre‐operative anaemia. Treatment of pre‐operative anaemia was hypothesised to reduce the associated risks and improve patient outcomes when undergoing elective major surgery [5].

To assess this hypothesis, the pre‐operative intravenous iron to treat anaemia in major surgery (PREVENTT) trial, a double‐blind, randomised controlled trial, was performed in patients with anaemia before elective, major, open abdominal surgery [6]. The primary analysis of the trial showed that that treatment with intravenous iron in all patients with pre‐operative anaemia increased haemoglobin concentration but did not reduce peri‐operative transfusion or mortality, nor did it impact patient outcomes during hospital stay.

The trial design was pragmatic according to normal NHS practice and included participants with anaemia defined by baseline haemoglobin concentration. At the time the study was conducted (2014–2018), pre‐operative iron status was not routinely assessed and additional testing for iron parameters to define inclusion into the trial was not feasible in the surgical pathways of the NHS in the UK [7]. Also, there is uncertainty as to the definitions of iron deficiency (absolute/functional/inflammatory iron deficiency or anaemia of chronic disease). To date, clinical trials assessing the efficacy of intravenous iron have had variable and broad inclusion criteria, including many without baseline iron parameters, i.e. just anaemia as the inclusion criteria [8].

To address this, as part of the PREVENTT protocol, in addition to routine local hospital blood testing, blood samples were collected for analysis in a central laboratory at randomisation before the administration of the study drug and again on the day of surgery [9]. Results from full blood count, serum ferritin and transferrin saturations (TSAT), were returned to the trial centre, but patients and staff at hospitals involved in the trial were blinded to the results of these blood tests. Predefined subgroup analysis was previously reported [6]. As part of the PREVENTT report, a detailed secondary analysis evaluating the interaction between type of iron deficiency anaemia and treatment effects seen within the PREVENTT trial was pre‐planned. This re‐analysis of the PREVENTT trial was based on baseline iron status (ferritin and TSAT), and red cell indices (mean corpuscular volume (MCV) and mean corpuscular haemoglobin (MCH)). The aim of this analysis was to determine whether specific iron deficiency phenotypes derived clinical benefits from intravenous iron before major abdominal surgery compared with placebo.

## Methods

The PREVENTT trial was a multicentre study in which adult patients with anaemia were allocated randomly 1:1 to receive 1000 mg of intravenous iron or placebo 10–42 days before major open abdominal surgery. Peri‐operative care was in concordance with local treatment pathways and the blood transfusion protocols were according to the NHS Blood and Transplant recommendations [10, 11]. The trial was approved by the UK National Research Ethics Committee for the East of England.

Full details of the protocol and results of the primary analysis have been reported previously [6]. The intervention group was administered a single dose of 1000 mg ferric carboxymaltose (Ferinject® Vifor Pharma, Zurich, Switzerland) in 100 ml saline. The control group was administered 100 ml saline. All patients, clinicians and research staff involved in the study were blinded to intervention status, with masked administration of the study drug via black tubing. The trial had two co‐primary outcomes: the composite endpoint of risk of blood transfusion or death; and the number of allogeneic blood transfusion episodes from randomisation until 30 days after the index operation.

Eligibility for the PREVENTT trial was determined in part by local laboratory results: patients were required to have a haemoglobin concentration ≥ 90 g.l^−1^ but ≤ 120 g.l^−1^ in women or 130 g.l^−1^ in men in the 4 weeks before randomisation. Additional blood samples were taken at randomisation before administration of the study drug and subsequently on the day of planned surgery for central laboratory analysis. These samples, EDTA (for a full blood count examination) and serum (for iron studies), were processed locally before courier transfer to a central laboratory for analysis (The Doctors Laboratory, London, UK: Sysmex xe‐2100 automated haematology system). Clinical and research staff and participants were blinded to the results.

For subgroup analyses of iron deficiency, patients were categorised as absolute iron deficiency (ferritin ≤ 30 μg.l^−1^), functional iron deficiency (ferritin 30–100 μg.l^−1^ or TSAT < 20%) and non‐iron deficient (ferritin > 100 μg.l^−1^ and TSAT > 20%). As there is no consensus on the definitions of absolute/functional/inflammatory iron deficiency or anaemia of chronic disease [8], the parameters were agreed by the Trial Steering Committee at the start of the trial and in the statistical analysis plan [6, 9]. Participants were also categorised on the basis of: serum ferritin (≤ 30 μg.l^−1^; 30–100 μg.l^−1^; ≥ 100 μg.l^−1^); TSAT (< 20% or ≥ 20%); MCV (< 80 fl or ≥ 80 fl); and MCH (< 27 pg or ≥ 27 pg).

To identify potential treatment effect(s) across subgroups for each of the primary and secondary outcomes, we used generalised linear models that included an interaction term between subgroups and treatment group. Those interactions were subsequently evaluated based on a log‐likelihood ratio test comparing the model with the interaction term to the nested model that excluded the interaction. The specific form of the generalised linear model used for each outcome corresponded to those published in the original paper [6]. Binomial regression was used to assess the co‐primary outcomes of risk of death or blood transfusion at 30 days following the index operation, postoperative complications, re‐admissions to hospital at 8 weeks and 6 months, and all‐cause mortality at 6 months with the effect reported as relative risks (RR). Negative binomial models offset for time at risk were used to assess the number of blood transfusions at 30 days following the index operation, and the total number of units of blood transfused (excluding large blood transfusions) at 30 days and 6 months, with effects reported as incidence rate ratios (IRR). Log‐linear models were used to assess ICU and hospital lengths of stay, with the effects reported as ratios of geometric means (RGM).

We also estimated an additional set of similar models, where the treatment effect was explored based on interactions between treatment arms and continuously measured biomarkers (haemoglobin, ferritin, TSAT, MCV, MCH) and where possible non‐linearity in the biomarker effects was accommodated using restricted cubic splines. Additional details for these models are given in online Supporting Information (Appendix S2).

All analyses were conducted with R (version 4.0.3; R Project for Statistical Computing, Vienna, Austria) and the RStudio interactive development environment (version 2022.02.3). Given the exploratory nature of the analyses, we did not adjust any reported p values for multiplicity.

## Results

In total, 487 participants were recruited across 46 hospitals in the UK from January 2014 to September 2018, of whom 244 were allocated to receive intravenous iron and 243 to receive placebo. For the co‐primary endpoints, 474 (97%) patients were included in the intention‐to‐treat analysis. The baseline characteristics of participants were well matched (Table 1). Complete central laboratory blood results were available for 452/487 (93%) participants overall (222/244 [91%] in the intravenous iron group and 230/243 [96%] in the placebo group).

**Table 1:** Baseline characteristics and surgical characteristics by baseline iron deficiency status. Values are mean (SD), number (proportion) or median (IQR [range]).

		Baseline iron deficiency status		
		Absolute	Functional	Normal
	n	n = 144	n = 225	n = 83
Age; y	452	56 (15)	66 (11)	66 (10)
Sex	452			
Female		97 (67%)	110 (49%)	46 (55%)
Baseline Hb; g.l^−1^	444	109 (100–116 [83–131])	112 (102–120 [63–142])	115 (108–123 [89–145])
ASA physical status	438			
1		39 (28%)	15 (6.8%)	5 (6.2%)
2		72 (53%)	149 (68%)	49 (60%)
3		26 (19%)	55 (25%)	27 (33%)
4		0	1 (0.5%)	0
5		0	0	0
Smoking history	450			
Never		80 (56%)	96 (43%)	36 (43%)
Ex		48 (34%)	111 (50%)	40 (48%)
Current		15 (10%)	17 (7.6%)	7 (8.4%)
Days between iron and surgery	452	15 (12–23 [8–205])	14 (12–20 [5–212])	15 (13–22 [7–113])
Underwent surgery	452	136 (94%)	219 (97%)	79 (95%)
Surgical time; min	175	125 (90–201 [27–830])	181 (126–300 [90–512])	249 (170–312 [78–472])
Anaesthetic time; min	325	182 (138–296 [0–685])	260 (180–385 [0–905])	294 (215–376 [65–615])

Baseline iron deficiency status: absolute iron deficiency (ferritin < 30 μg.l^−1^); functional iron deficiency (ferritin between 30 and 100 μg.l^−1^ OR TSAT < 20%); normal iron levels.

Most patients had iron deficiency (369/452 [82%]) at randomisation, one‐third had absolute iron deficiency (144/452 [32%]) and half had functional iron deficiency (225/452 [50%]). Only 18% of participants (83/452) were anaemic despite being apparently iron replete; that is, having a serum ferritin ≥ 100 μg.l^−1^ and TSAT ≥ 20% at randomisation. At randomisation, laboratory variables for iron deficiency and red blood cell indices were similar in the placebo and intravenous iron groups (Table 1).

**Figure 1:**
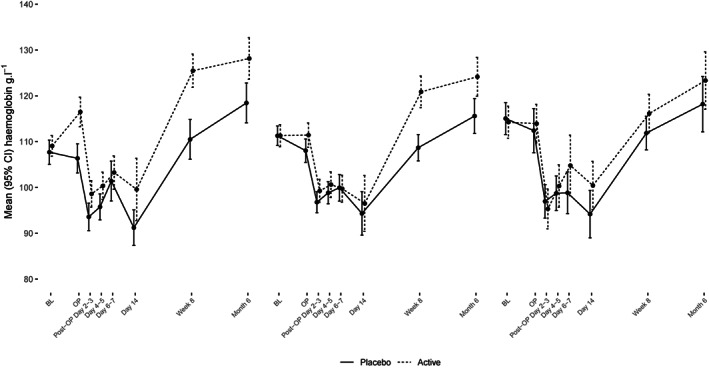
Haemoglobin concentration in patients allocated randomly to intravenous iron or placebo, subdivided into absolute iron deficiency (ferritin < 30 μg.l^−1^); functional iron deficiency (ferritin between 30 and 100 μg.l^−1^ OR TSAT < 20%); and normal iron levels. BL, baseline; OP, operation.

The efficacy of intravenous iron on haemoglobin concentration was greatest in those patients with absolute iron deficiency; average haemoglobin increased significantly in the intravenous iron group compared with placebo by the time of surgery, mean difference 8.9 g.l^−1^, 95%CI 5.3–12.5. A moderate rise in haemoglobin concentration was seen in those with functional iron deficiency anaemia, mean difference 2.8 g.l^−1^, 95%CI 0.01–5.7. There was little change seen in those patients who were non‐iron deficient (Fig. 1).

Re‐analysis of the PREVENTT trial by baseline iron status did not impact initial conclusions with respect to the co‐primary endpoints of the trial. There was no difference between treatment groups for risk of death or any transfusion in 30 days in patients with either absolute iron deficiency (ferritin < 30 μg.l^−1^; 17/69 placebo, 14/75 intravenous iron; RR 0.76, 95%CI 0.40–1.42; p = 0.39); or functional iron deficiency (ferritin 30–100 μg.l^−1^ or TSAT < 20%; 58/193 placebo, 53/176 intravenous iron; RR 1.0, 95% CI 0.73–1.37; p = 0.99); and no suggestion of effect by subgroup (p values for interaction 0.33 and 0.45, respectively). There was no statistically significant difference between groups for the number of transfusions within 30 days in patients with either absolute iron deficiency mean (SD) (0.5 (1.1) placebo vs. 0.2 (0.5) intravenous iron; IRR 0.48, 95% CI 0.21–1.07; p = 0.07) or functional iron deficiency (0.5 (1.0) placebo vs. 0.5 (1.0) intravenous iron; IRR 0.95, 95% CI 0.63–1.43; p = 0.8), and no suggestion of effect by subgroup (p values for interaction 0.06 and 0.29, respectively) (Tables 2 and 3).

**Table 2:** Subgroup analysis for death or any transfusion within 30 days. Values are number (proportion).

Subgroups	Placebo	Active	Estimated effect: RR (95%CI)*	LRT P**
Ferritin < 30 μg.l^−1^	17/69 (25%)	14/75 (19%)	0.76 (0.4–1.42); p = 0.39	0.33
Ferritin 30–100 μg.l^−1^	17/63 (27%)	20/53 (38%)	1.4 (0.82–2.38); p = 0.22	0.33
Ferritin ≥ 100 μg.l^−1^	32/98 (33%)	31/94 (33%)	1.01 (0.67–1.51); p = 0.96	0.33
TSAT < 20%	55/174 (32%)	49/163 (30%)	0.95 (0.69–1.31); p = 0.76	0.13
TSAT ≥ 20%	8/50 (16%)	15/53 (28%)	1.77 (0.82–3.81); p = 0.14	0.13
Ferritin < 100 μg.l^−1^OR TSAT < 20%	58/193 (30%)	53/176 (30%)	1 (0.73–1.37); p = 0.99	0.45
Ferritin ≥ 100 μg.l^−1^AND TSAT ≥ 20%	7/35 (20%)	12/43 (28%)	1.4 (0.62–3.16); p = 0.42	0.45
MCH < 27 pg	28/77 (36%)	21/75 (28%)	0.77 (0.48–1.23); p = 0.27	0.12
MCH ≥ 27 pg	38/150 (25%)	46/148 (31%)	1.23 (0.85–1.77); p = 0.27	0.12
MCV < 80 fl	24/57 (42%)	9/44 (20%)	0.49 (0.25–0.94); p = 0.03	0.01
MCV ≥ 80 fl	42/169 (25%)	58/179 (32%)	1.3 (0.93–1.83); p = 0.12	0.01

LRT, likelihood ratio test; TSAT, transferrin saturation; MCH, mean corpuscular haemoglobin; MCV, mean corpuscular volume.

* Indicates p values for the within‐subgroup estimate of the treatment effect.

** Indicates p values from the likelihood ratio tests of the interaction between subgroup and study group.

**Table 3:** Subgroup analysis for the number of transfusions within 30 days. Values are mean (SD).

Subgroups	Placebo	Active	Estimated effect: IRR (95%CI)*	LRT P**
Ferritin < 30 μg.l^−1^	0.5 (1.1)	0.2 (0.5)	0.48 (0.21–1.07); p = 0.07	0.06
Ferritin 30–100 μg.l^−1^	0.4 (0.9)	0.7 (1.3)	1.56 (0.75–3.23); p = 0.23	0.06
Ferritin ≥ 100 μg.l^−1^	0.5 (0.8)	0.5 (0.9)	1.07 (0.65–1.78); p = 0.78	0.06
TSAT < 20%	0.5 (1.0)	0.5 (1.0)	0.92 (0.6–1.4); p = 0.7	0.29
TSAT ≥ 20%	0.3 (0.7)	0.4 (0.7)	1.55 (0.63–3.8); p = 0.34	0.29
Ferritin < 100 μg.l^−1^OR TSAT < 20%	0.5 (1.0)	0.5 (1.0)	0.95 (0.63–1.43); p = 0.8	0.29
Ferritin ≥ 100 μg.l^−1^AND TSAT ≥ 20%	0.3 (0.6)	0.4 (0.8)	1.72 (0.66–4.51); p = 0.27	0.29
MCH < 27 pg	0.6 (1.0)	0.4 (0.9)	0.66 (0.36–1.19); p = 0.17	0.11
MCH ≥ 27 pg	0.4 (0.8)	0.5 (1.0)	1.25 (0.77–2); p = 0.37	0.11
MCV < 80 fl	0.7 (1.1)	0.3 (1.0)	0.47 (0.21–1.02); p = 0.06	0.03
MCV ≥ 80 fl	0.4 ± 0.8	0.5 (0.9)	1.26 (0.82–1.94); p = 0.28	0.03

LRT, likelihood ratio test; IRR, incidence rate ratio; TSAT, transferrin saturation; MCH, mean corpuscular haemoglobin; MCV, mean corpuscular volume.

* Indicates p values for the within‐subgroup estimate of the treatment effect.

** Indicates p values from the likelihood ratio tests of the interaction between subgroup and study group.

Overall, the rate of major complications (defined as Clavien–Dindo classification grade 3 or above) in patients following major open abdominal surgery was relatively low (46 participants (9.4%)) with no significant differences seen between the subgroups (Table 4). However, patients with absolute iron deficiency had a longer length of hospital stay in the intravenous iron group (Table 5) (mean (SD) 8.1 (1.8) days), compared with placebo (mean (SD) 6.3 (1.9) days), RGM 1.28, 95%CI 1.05–1.56; p = 0.02. There was no difference in patients with functional iron deficiency (mean (SD) 8.1 (2.1) days in the placebo group and mean (SD) 9.3 (2.1) days in the intravenous iron group; RGM 1.14, 95% CI 0.99–1.32; p = 0.07) (Table 5).

**Table 4:** Subgroup analysis for any Clavien‐Dindo 3+ postoperative complications. Values are number (proportion).

Subgroups	Placebo	Active	Estimated effect: RR (95%CI)*	LRT P**
Ferritin < 30 μg.l^−1^	2/65 (3%)	4/73 (5%)	1.78 (0.34–9.4); p = 0.5	0.03
Ferritin 30–100 μg.l^−1^	4/62 (6%)	8/53 (15%)	2.34 (0.75–7.34); p = 0.14	0.03
Ferritin ≥ 100 μg.l^−1^	18/95 (19%)	8/93 (9%)	0.45 (0.21–0.99); p = 0.05	0.03
TSAT < 20%	19/167 (11%)	17/161 (11%)	0.93 (0.5–1.72); p = 0.81	0.51
TSAT ≥ 20%	5/49 (10%)	3/52 (6%)	0.57 (0.14–2.24); p = 0.42	0.51
Ferritin < 100 μg.l^−1^OR TSAT < 20%	19/185 (10%)	17/173 (10%)	0.96 (0.51–1.78); p = 0.89	0.37
Ferritin ≥ 100 μg.l^−1^AND TSAT ≥ 20%	5/35 (14%)	3/43 (7%)	0.49 (0.13–1.9); p = 0.3	0.37
MCH < 27 pg	4/76 (5%)	7/74 (9%)	1.8 (0.55–5.88); p = 0.33	0.13
MCH ≥ 27 pg	20/143 (14%)	13/146 (9%)	0.64 (0.33–1.23); p = 0.18	0.13
MCV < 80 fl	2/57 (4%)	5/44 (11%)	3.24 (0.66–15.91); p = 0.15	0.04
MCV ≥ 80 fl	22/161 (14%)	15/176 (9%)	0.62 (0.34–1.16); p = 0.14	0.04

LRT, likelihood ratio test; TSAT, transferrin saturation; MCH, mean corpuscular haemoglobin; MCV, mean corpuscular volume.

* Indicates p values for the within‐subgroup estimate of the treatment effect.

** Indicates p values from the likelihood ratio tests of the interaction between subgroup and study group.

**Table 5:** Subgroup analysis for hospital length of stay. Values are mean (SD).

Subgroups	Placebo	Active	Estimated effect: RGM (95%CI)*	LRT P**
Ferritin <30 μg.l^−1^	6.3 (1.9)	8.1 (1.8)	1.28 (1.05–1.56); p = 0.02	<0.01
Ferritin 30–100 μg.l^−1^	8.2 (2.1)	11.5 (2.2)	1.39 (1.06–1.84); p = 0.02	<0.01
Ferritin ≥100 μg.l^−1^	10.5 (2.0)	9.1 (1.8)	0.87 (0.72–1.05); p = 0.14	<0.01
TSAT <20%	8.3 (2.1)	9.1 (2.0)	1.09 (0.94–1.27); p = 0.25	0.96
TSAT ≥20%	8.7 (2.0)	9.6 (1.8)	1.1 (0.86–1.42); p = 0.45	0.96
Ferritin <100 μg.l^−1^ OR TSAT <20%	8.1 (2.1)	9.3 (2.0)	1.14 (0.99–1.32); p = 0.07	0.13
Ferritin ≥100 μg.l^−1^ AND TSAT ≥20%	10.5 (1.8)	9.2 (1.8)	0.88 (0.68–1.14); p = 0.33	0.13
MCH < 27 pg	6.3 (2.0)	8.2 (1.8)	1.3 (1.06–1.6); p = 0.01	0.04
MCH ≥27 pg	10 (2.0)	9.9 (2.0)	0.99 (0.85–1.16); p = 0.88	0.04
MCV < 80 fl	6.6 (1.9)	8.5 (1.9)	1.29 (1.00–1.66); p = 0.06	0.11
MCV ≥80 fl	9.4 (2.0)	9.5 (1.9)	1.01 (0.87–1.17); p = 0.92	0.11

LRT, likelihood ratio test; RGM, ratio of geometric means; TSAT, transferrin saturation; MCH, mean corpuscular haemoglobin; MCV, mean corpuscular volume.

* Indicates p values for the within‐subgroup estimate of the treatment effect.

** Indicates p values from the likelihood ratio tests of the interaction between subgroup and study group.

In supplemental analyses of potential treatment effect as a function of continuously measured variables from MCH and iron studies (ferritin, transferrin saturations), these values did not influence the co‐primary endpoints of the trial. Participants with an MCV < 80 fl who received intravenous iron had a reduced likelihood of death or blood transfusion at 30 days (RGM 0.49, 95% CI 0.25–0.94; p = 0.03), see online Supporting Information (Appendix S2 and Figs. S1–S5). Additionally, patients with lower MCV values tended to receive fewer units of blood following the administration of intravenous iron compared with the placebo group (see online Supporting Information, Figure S4).

## Discussion

The PREVENTT trial showed that the use of intravenous iron compared with placebo in patients with anaemia before major abdominal surgery did not affect peri‐operative outcomes. Predefined, re‐analysis of data by baseline markers of iron deficiency confirmed that most patients (82%) had iron deficiency at inclusion meaning the trial was adequately powered (> 80% power with a 5% α) for iron deficiency anaemia to the co‐primary endpoints. These analyses show that intravenous iron compared with placebo increased pre‐operative haemoglobin levels most effectively in patients with absolute iron deficiency (ferritin < 30 μg.l^−1^), less so in those with functional iron deficiency (ferritin 30–100 μg.l^−1^ or TSAT < 20%) and was not effective in patients who were non‐iron deficient. However, the treatment effect to increase haemoglobin levels before surgery did not translate into clinical benefit in terms of reduced need for blood transfusion or death, the amount of blood received, postoperative complications or length of hospital stay.

The accurate diagnosis of iron deficiency remains challenging, especially in the peri‐operative setting. Serum ferritin is critical in defining absolute iron deficiency. However, ferritin can be elevated due to its role as an acute phase protein during inflammation, due to the illness for which the patient is undergoing surgery (often malignancy) or patient comorbidities such as diabetes, renal or cardiac disease [12]. Similar diagnostic limitations are also seen with the use of transferrin saturations and there is no consensus on the definitions of absolute/functional/inflammatory iron deficiency or anaemia of chronic disease [8].

Nevertheless, the use of intravenous iron in surgical patients, which is thought to bypass hepcidin‐mediated down‐regulation of ferroportin to stimulate erythropoiesis, has been seen to be effective to treat anaemia in a range of conditions and situations including in patients on ICU where baseline ferritin and TSAT are often very high [13]. The PREVENTT trial confirms the effectiveness of intravenous iron to increase haemoglobin levels in patients with pre‐operative absolute iron deficiency defined as ferritin < 30 μg.l^−1^ or those with significant hypochromia (MCV < 80 fl). There may be a clinical effect with reduction in the risk of the co‐primary endpoint (mortality or transfusion at 30 days postoperatively), although statistical significance was lost when considering the risk of transfusion in isolation.

The identification and management of pre‐operative anaemia and iron deficiency has now become recommended as part of routine care [14, 15, 16]. While the associations between pre‐operative anaemia and worse postoperative outcomes are well established, the assumption was that the underlying causality was iron deficiency in most cases, and that it could be rectified by administering pre‐operative intravenous iron. This assumption of the cause of anaemia was confirmed in PREVENTT, where although the inclusion, for practical reasons, was anaemia, most patients (82%) had iron deficiency anaemia [5, 8, 12]. However, these repeated analyses show that, despite correction of pre‐operative iron deficiency anaemia, this did not translate into clinical benefit.

The findings from PREVENTT and this re‐analysis have important implications for clinical practice for patients undergoing surgery, specifically in patients with iron deficiency anaemia. The data should also be of reassurance to clinicians who held reservations about the validity and generalisability of the PREVENTT findings given the diagnostic limitation of pre‐operative iron deficiency and the generalised administration of intravenous iron. Further investigation to accurately define iron deficiency in the pre‐operative period and the role of inflammation may be helpful in the identification of patients who might benefit from such interventions. The results should also provide reassurance to patients and staff in the last 2 years of COVID‐19 lockdown, that patients have not been disadvantaged by not receiving pre‐operative management of anaemia with intravenous iron [17].

The link between pre‐operative anaemia and adverse patient outcomes has been shown in large cohort studies. However, it should be noted that those patients with pre‐operative anaemia were often older, had more comorbidities and were sicker [1]. Therefore, although statistically addressed in multivariable regression modelling, the effect of pre‐operative anaemia on patient outcomes is associative. Statistical analyses (often on retrospective datasets) do not address whether pre‐operative anaemia was causal to increased peri‐operative patient risk. Indeed, pre‐operative anaemia could simply be a marker of overall patient risk. In PREVENTT, these confounding factors of patient age, comorbidities and risk were well balanced by the randomised trial design between the placebo and intervention groups.

The use of intravenous iron to treat pre‐operative anaemia has been advocated as part of patient blood management [18]. In PREVENTT, the blood transfusion protocol followed the national guidelines from NHS Blood and Transplant and variation from these was balanced by the effect of randomisation. However, it should be noted that patient blood management is a patient‐centred, systematic, evidence‐based approach to improve patient outcomes by managing and preserving a patient's own blood, while promoting patient safety and empowerment [18]. Specifically, the aim of patient blood management is not to correct a laboratory number (i.e. increase haemoglobin) but to improve patient outcomes. Similarly, the focus on anaemia correction and blood transfusion in isolation is a less important clinical trial (or clinical reality) endpoint relative to patient‐centred outcomes. In this regard, PREVENTT also confirms the findings seen in a recent large single centre study by Spahn et al. in cardiac surgery where, with high transfusion rates, the use of a generic combination of iron, erythropoietin stimulating drug, vitamin B_12_ and folic acid, a lower rate of blood transfusion was seen compared with placebo, but failed to show impact on any patient‐specific outcomes or hospital length of stay [19].

Despite intravenous iron for the treatment of pre‐operative iron deficiency anaemia being promulgated as ‘standard of care’ by national and international best practice guidelines [14, 15, 16], the randomised trial evidence that supported its use before the publication of PREVENTT was limited. Indeed, an updated systematic review and meta‐analysis in 2019 by Ng et al. concluded that “*the use of iron therapy for pre‐operative anaemia does not show a statistically significant reduction in the proportion of patients who received an allogeneic blood transfusion compared to no iron therapy*” [20]. It might, therefore, be argued that until data supportive of the use of intravenous iron for the treatment of pre‐operative anaemia are produced, this practice represents low value care [21], and should not be administered outside of a clinical trial.

The strengths of this analysis are those of the PREVENTT trial; specifically, allocation concealment, double‐blinding and placebo control. Additionally, a very high proportion (93%) of the total cohort were able to be included in this subsequent analysis on iron deficiency anaemia, so high was the level of adherence to the trial protocol. The wide range of different definition iron statuses in included patients (including those with a pre‐operative iron deficiency anaemia with serum ferritin < 30 μg.l^−1^) means the findings will be generalisable and applicable to most patients who present for elective, major abdominal surgery.

We acknowledge the limitations of secondary analyses and urge caution when interpreting the results of subgroup analyses following an overall null finding [22]. Like most randomised trials, PREVENTT was designed to reliably detect the smallest clinically important treatment effect of pre‐operative intravenous iron on patient outcomes in an overall sample of recruited patients, and to assess if the intervention should be promoted as standard of care to all patients with pre‐operative anaemia and iron deficiency. Thus, by design, PREVENTT was not designed to identify any specific subgroup or interactions in which there may be an effect. Therefore, failure to detect a treatment effect in this context cannot exclude the existence of one [23]. At the same time, by performing such a comprehensive examination, issues with multiplicity arise, and we should be similarly cautious to not over‐interpret the few relatively small statistically significant findings when interpreting the interactions or the within‐subgroup treatment effects. This is relevant to the findings that intravenous iron increased length of hospital stay in patients with absolute iron deficiency and increased the risk of major postoperative complications when serum ferritin was > 100 μg.l^−2^.

When considering the subgroup of patients with absolute iron deficiency, defined as ferritin < 30 μg.l^−1^ or significant hypochromia (MCV < 80 fl), we maintain that this group of patients should receive treatment (including intravenous iron) regardless of the reason for hospitalisation (medical or surgical). All patients with absolute iron deficiency should be investigated and treated according to local guidelines no matter if they are pre‐ or postoperative or have another cause (medical or gynaecological). It may be that pre‐assessment clinics are an opportune and appropriate setting to screen for such patients.

The PREVENTT trial answers two important questions. Firstly, should all patients with anaemia receive intravenous iron before surgery? These results suggest that the answer is ‘no’. Secondly, should patients with iron deficiency and anaemia receive intravenous iron in advance of their surgery to improve their peri‐operative outcome? These data suggest that the answer is also ‘no’. The central message of PREVENTT, therefore, is not that those patients with iron deficiency anaemia should not be treated’ merely that delaying their surgery to undertake this intervention does not appear to be necessary. The dogma supporting the universal administration of intravenous iron before major abdominal surgery must now be questioned, at least until such time that its proponents are able to produce supportive randomised trial data of similar quality to PREVENTT.

However, the impact of postoperative anaemia was recently highlighted in a large prospective study as associated with increased risk of unplanned patient re‐admission within 30 days of hospital discharge [24]. It is unclear whether the use of intravenous iron can improve recovery in patients after major abdominal surgery (with associated blood loss) as indicated by the novel findings in the PREVENTT trial that the intravenous iron group had a higher increased haemoglobin level after discharge from hospital associated with a significant reduction in re‐admissions for complications. The quality of data in the postoperative setting are comparable with the association of pre‐operative anaemia and adverse patient outcomes at operation that triggered the PREVENTT study. These associations and suggestion of a potential treatment effect from intravenous iron on postoperative outcomes should be formally assessed in a subsequent clinical trial.

To conclude, the findings of this analysis on the use of intravenous iron compared with placebo in patients with pre‐operative iron deficiency anaemia support the validity and generalisability of the PREVENTT trial results.

## Supporting information


**Appendix S1.** The PREVENTT trial collaborators.

**Appendix S2.** Models with interactions based on continuously measured laboratory iron biomarkers.
**Figure S1.** First co‐primary outcome – death or any transfusion within 30 days.
**Figure S2.** Second primary outcome – Number of transfusions at 30 days.
**Figure S3.** Clavien–Dindo grade 3 or above to discharge.
**Figure S4.** Units of blood to 30 days.
**Figure S5.** Hospital length of stay.
