# Supplementary material for: The association between iron deficiency and outcomes: a secondary analysis of the intravenous iron therapy to treat iron deficiency anaemia in patients undergoing major abdominal surgery (PREVENTT) trial
Source: Anaesthesia. 2022 Dec 8;78(3):320–9. doi: 10.1111/anae.15926 (PMC10107684; doi:10.1111/anae.15926)
Supplement: Supplementary file 1 — Appendix S1. The PREVENTT trial collaborators. [file ANAE-78-320-s001.docx]

**Appendix S1 PREVENTT Study team, contributors and collaborators**

**PREVENTT ID Writing Committee**

Toby Richards (Chief Investigator), Martin Besser, John Browne, Ben Clevenger, Anastazia Kegan, Andrew Klein, Lachlan Miles, Iain MacDougall, Ravishankar Rao Baikady

**Trial Statistician**

Darren Dahly

**Blood Sample Analysis**

The Doctors Laboratory

**PREVENTT Trial contributors**

**Trial Steering Committee**

Andrew Bradbury (Chair), Toby Richards (Chief Investigator), Trevor Burley, Shelley Van Loen, Stefan Anker, Andrew Klein, Iain MacDougall, Gavin Murphy, Martin Besser and Isabel Unsworth.

Observers - Tim Clayton, Tim Collier, Kimberley Potter, Sandy Abeysiri, Richard Evans, Rosemary Knight, Rebecca Swinson, Laura Van Dyck and Jane Keidan.

**Data Safety and Monitoring Committee**

Lorna Williamson (Chair), Angela Crook, and John Pepper

Unblinded statisticians supporting the DSMC were Joanna Dobson, Simon Newsome, Tom Godec and Matthew Dodd.

**Project Management Group**

Toby Richards, Laura Van Dyck, Richard Evans, Sandy Abeysiri, Ben Clevenger, Anna Butcher, Rebecca Swinson, Tim Collier and Kimberley Potter.

**Study Design and Support**

Stefan Anker, John Kelly, Steven Morris, John Browne, Jane Keidan, Michael Grocott, Marisa Chau and Rosemary Knight.

**Trial Statistician**

Timothy Collier

**Randomisation Service**

Sealed Envelope^TM^

**Blood Sample Analysis**

The Doctors Laboratory

## Contributing Sites & Investigators

Royal Marsden NHS Foundation Trust *(118 patients, opened April 2, 2014)*

Ravishankar Rao Baikady (PI), Ethel Black, Helen Lawrence, Maria Kouthra, Katherine Horner, Sham Jhanji, Ed Todman, Zoe Keon-Cohen, Martin Rooms, Judith Tomlinson, Ian Bailes, Susanna Walker, Katrina Pirie, Michelle Gerstman, Ramanathan Kasivisvanathan, Sophie Uren, David Magee, Alex Eeles, Rob Anker, Jamie McCanny, Michelle O’Mahony, Toby Reynolds, Sian Batley, Aoife Hegarty, Simon Trundle, Francesca Mazzola, Kate Tatham, Alina Balint, Ben Morrison, Matthew Evans, Ching Ling Pang, Lorna Smith, Charlotte Wilson, Victoria Sjorin, Poonam Khatri, Marco Wilson, David Parkinson

University College London Hospitals NHS Foundation Trust *(38 patients, opened September 1, 2013)*

James Crosbie (PI), Khaled Dawas (PI), Deborah Smyth, Georgia Bercades, Jung Ryu, Anna Reyes, Gladys Martir, Laura Gallego, Alison Macklin, Magda Rocha, Dr Karen Tam, Dr David Brealey

Guy's and St Thomas' NHS Foundation Trust *(25 patients, opened May 7, 2014)*

Jugdeep Dhesi (PI), Catriona Morrison, Joanna Hardwick, Jude Partridge, Philip Braude, Andrew Rogerson, Nymah Jahangir, Clare Thomson, Lizzie Biswell, Jason Cross, Ffion Pritchard, Aminata Mohammed, Deirdre Wallace, Ma Gem Galat, Jane Okello, Rebecca Symes, Mollika, Chakravorty, Josette Leon, Angela Cape (pharmacy), Charlotte Gibbs (pharmacy)

Sheffield Teaching Hospitals NHS Foundation Trust *(24 patients, opened November 11, 2013)*

Sumayer Sanghera (PI), Andy Dennis, Faith Kibutu, Joyce Fofie, Sarah Bird, Abiola Alli, Yvonne Jackson

North Bristol NHS Trust *(23 patients, opened February 4, 2014)*

Salah Albuheissi (PI), Carol Brain, Connie Shiridzinomwa

Royal Cornwall Hospitals NHS Trust *(23 patients, opened September 19, 2013)*

Catherine Ralph (PI), Belinda Wroath, Fiona Hammonds, Benita Adams, John Faulds, Sara Staddon

King's College Hospital NHS Foundation Trust *(18 patients, opened February 26, 2016)*

Timothy Hughes (PI), Sian Saha, Clare Finney, Clair Harris, Clare Mellis, Lucy Johnson, Paul Riozzi, Adam Yarnold, Fraser Buchanan, Philip Hopkins, Louise Greig, Harriet Noble

University Hospital Southampton NHS Foundation Trust *(18 patients, opened January 7, 2014)*

Mark Edwards (PI), Mike Grocott, James Plumb, David Harvie, Ahilanandan Dushianthan, Mai Wakatsuki, Samantha Leggett, Karen Salmon, Clare Bolger, Rachel Burnish, James Otto, Gurinder Rayat, Kim Golder, Pauline Bartlett, Sitara Bali, Leanne Seaward, Beverley Wadams, Bryony Tyrell, Hannah Collins, Natasha Tantony, Rosie Geale, Amber Wilson, Darran Ball

Oxford University Hospitals NHS Foundation Trust *(15 patients, opened October 22, 2015)*

Ian Lindsey (PI), Debbie Barker, Madeleine Thyseen

South Tees Hospitals NHS Foundation Trust *(11 patients, November, 20, 2014)*

Patrick Chiam (PI), Carol Hannaway, Kerry Colling

The Hillingdon Hospitals NHS Foundation Trust *(11 patients, opened October 4, 2013)*

Cheryl Messer (PI), Neil Verma, Mariam Nasseri, Gail Poonawala, Abbie Sellars, James Harris (PI), Pratyuja Mainali

Mid Essex Hospital Services NHS Trust *(10 patients, opened March 11, 2015)*

Toby Hammond (PI), Al Hughes, David O'Hara, Fiona McNeela, Lauren Shillito

Leeds Teaching Hospitals NHS Trust *(10 patients, opened November 12, 2014)*

Alwyn Kotze (PI), Catherine Moriarty

York Teaching Hospital NHS Foundation Trust *(10 patients, opened December 17, 2013)*

Jonathan Wilson (PI), Simon Davies, David Yates, Joe Carter, Jon Redman, Sara Ma, Kate Howard, Heidi Redfearn, Danielle Wilcock

Imperial College Healthcare NHS Trust *(9 patients, opened November 6, 2015)*

Justine Lowe (PI), Asela Dharmadasa (PI), Tamara Alexander, Jasmine Jose, Gillian Hornzee, Fatima Akbar, Severine Rey, Anoop Patel, Samantha Coulson, Rajan Saini, Joseph Santipillai, Thomas McCretton, Ian Bailes (PI), Jamie McCanny, Kiran Chima, Karen Collins, Byiravey Pathmanathan, Anjalee Chattersingh, Laura McLeavy, Zayneb Al-Saadi

Salford Royal NHS Foundation Trust *(9 patients, opened November 17, 2014)*

Manju Patel (PI), Sofia Skampardoni, Rajkumar Chinnadurai, Vicky Thomas, Anne Keen, Katherine Pagett, Clare Keatley, Jason Howard, Leigh Willoughby (PI), Marie Greenhalgh

The Dudley Group NHS Foundation Trust *(8 patients, opened March 8, 2016)*

Stephen Jenkins (PI), Ranjit Gidda, Angela Watts

Royal Liverpool and Broadgreen University Hospitals NHS Trust *(8 patients, opened February 20, 2015)*

Chris Breaton (PI), Jane Parker

Royal Free London NHS Foundation Trust *(8 patients, opened December 19, 2013)*

Susan Mallett (PI), Sarah James

Wye Valley NHS Trust *(7 patients, opened May 20, 2015)*

Lisa Penny (PI), Kim Chan, Tamsin Reeves, Marisa Catterall, Sue Williams, Janine Birch, Kate Hammerton, Nicola Williamson, Anni Thomas, Melanie Evans, Lily Mercer, Gill Horsfield, Claire Hughes

Blackpool Teaching Hospitals NHS Foundation Trust *(7 patients, opened September 1, 2014)*

Jason Cupitt (PI), Emma Stoddard

Liverpool Women's NHS Foundation Trust *(6 patients, May 4, 2018)*

Helen McNamara (PI), Chloe Birt

North West Anglia NHS Foundation Trust (Peterborough) *(6 patients, opened December 12, 2015)*

Alexander Hardy (PI), Robert Dennis, Deborah Butcher, Susie O’Sullivan, Alan Pope

University Hospital of South Manchester NHS Foundation Trust *(6 patients, opened September 29,2014)*

Sumaya Elhanash (PI), Stephen Preston, Helen Officer, Andrea Stoker, Stuart Moss, Alison Walker, Anna Gipson, Julie Melville, Joanne Bradley-Potts, Richard McCormac, Vivienne Benson, Kirsty Melia, Julie Fielding, Wendy Guest

Swansea Bay University Health Board *(6 patients, opened July 8, 2014)*

Simon Ford (PI)

Gloucestershire Hospitals NHS Foundation Trust *(5 patients, opened February 22, 2018)*

Henry Murdoch (PI), Susan Beames, Paula Townshend, Kayleigh Collins, Jon Glass, Bethan Cartwright

Aintree University Hospital NHS Foundation Trust *(5 patients, opened March 11, 2016)*

Balsam Altemimi (PI), Lucy Berresford

Royal Surrey County Hospital NHS Foundation Trust *(5 patients, opened September 19, 2014)*

Chris Jones (PI), Leigh Kelliher, Sam de Silva, Katie Blightman

Central Manchester University Hospitals NHS Foundation Trust *(5 patients, opened Juner 27, 2014)*

Kate Pendry (PI), Lebina Pinto

Barts Health NHS Trust *(5 patients, opened May 9, 2014)*

Shubha Allard (PI), Louise Taylor

The Newcastle upon Tyne Hospitals NHS Foundation Trust *(5 patients, opened March 7, 2014)*

Ahmed Chishti (PI), Julia Scott

Norfolk and Norwich University Hospitals NHS Foundation Trust *(4 patients, opened November 6, 2015)*

Debbie O'Hare (PI), Michael Lewis

Pennine Acute Hospitals NHS Trust *(4 patients, July 22, 2015)*

Zahid Hussain (PI), Karen Hallett, Susan Dermody, Carolyn Corbett, Louise Morby

Dorset County Hospital NHS Foundation Trust *(4 patients, opened December 6, 2013)*

Matthew Hough (PI), Sarah Williams, Patricia Williams, Sarah Horton, Pauline Ashcroft, Anthony Homer

Royal Edinburgh Hospital - NHS Lothian *(3 patients, opened March 4, 2016)*

Alastair Lang, Heidi Dawson, Ewen Harrison (PI)

Royal Devon and Exeter NHS Foundation Trust *(3 patients, opened May 12, 2014)*

John Thompson (PI)

North West Anglia NHS Foundation Trust (Hinchingbrook) *(1 patient, opened February 1,2018)*

Vimal Hariharan (PI), Vanessa Goss

Basildon and Thurrock University Hospitals NHS Foundation Trust *(1 patient, opened October 4, 2017)*

Ramachandran Ravi (PI), Georgina Butt, Mark Vertue

Nottingham University Hospitals NHS Trust *(1 patient, opened May 27, 2016)*

Austin Acheson (PI), Oliver Ng, Debbie Bush, Edward Dickson, Amy Ward

Brighton and Sussex University Hospitals NHS Trust *(1 patient, opened March 7, 2014)*

Sophie Morris (PI), R&D Management

Maidstone and Tunbridge Wells NHS Trust *(1 patient, opened October 10, 2013)*

Andrew Taylor (PI), Rebecca Casey

Countess of Chester Hospital NHS Foundation Trust *(0 patients, opened April 26, 2017)*

Lawrence Wilson (PI), Dale Vimalachandran, Maria Faulkner, Helen Jeffrey, Claire Gabrielle, Sharon Martin

Gateshead Health NHS Foundation Trust *(0 patients, opened November, 232015)*

Andrew Bracewell (PI), Jenny Ritzema, David Sproates

Northampton General Hospital NHS Trust *(0 patients, opened December 22, 2014)*

Farhad Alexander-Sefre (PI)

West Suffolk NHS Foundation Trust *(0 patients, opened September 16, 2014)*

Christiane Kubitzek (PI), Sally Humphreys, James Curtis, Paul Oats, Sandra Swann, Abbie Holden, Claire Adam

University Hospitals Bristol NHS Foundation Trust *(0 patients, opened May 19, 2014)*

Louise Flintoff, Claudia Paoloni (PI), Karen Bobruk
