# Supplementary material for: The association between iron deficiency and outcomes: a secondary analysis of the intravenous iron therapy to treat iron deficiency anaemia in patients undergoing major abdominal surgery (PREVENTT) trial
Source: Anaesthesia. 2022 Dec 8;78(3):320–9. doi: 10.1111/anae.15926 (PMC10107684; doi:10.1111/anae.15926)
Supplement: Supplementary file 2 — Appendix S2. Models with interactions based on continuously measured laboratory iron biomarkers. Figure S1. First co‐primary outcome – death or any transfusion within 30 days. Figure S2. Second primary outcome – Number of transfusions at 30 days. Figure S3. Clavien–Dindo grade 3 or above to discharge. Figure S4. Units of blood to 30 days. Figure S5. Hospital length of stay. [file ANAE-78-320-s002.docx]

**Appendix S2** Models with interactions based on continuously measured lab iron biomarkers

To identify potential treatment effect(s) across subgroups for each of the primary and secondary outcomes, we used generalised linear models (GLM) that included an interaction term between continuously measured markers of iron status and treatment arm. Those interactions were subsequently evaluated based on a log-likelihood ratio test (LRT) comparing the model with the interaction term to the nested model that excluded the interaction. Logistic regression was used to assess the co-primary outcomes of risk of death or blood transfusion at 30 days following the index operation, postoperative complications, readmissions to hospital at eight weeks and six months, and all-cause mortality at six months; quasi-Poisson models offset for time at risk were used to assess the number blood transfusions at 30 days following the index operation, and the total number of units of blood transfused (excluding large blood transfusions) at 30 days and six months. Quasi-Poisson models with no offset were used to assess ICU and hospital lengths of stay. Models results are reported by plotting the treatment arm specific predictions as a function of each of the continuously measured biomarkers, accompanied by tables with the LRT p-values.

#### Figure S1. First co-primary outcome - death or any transfusion within 30 days


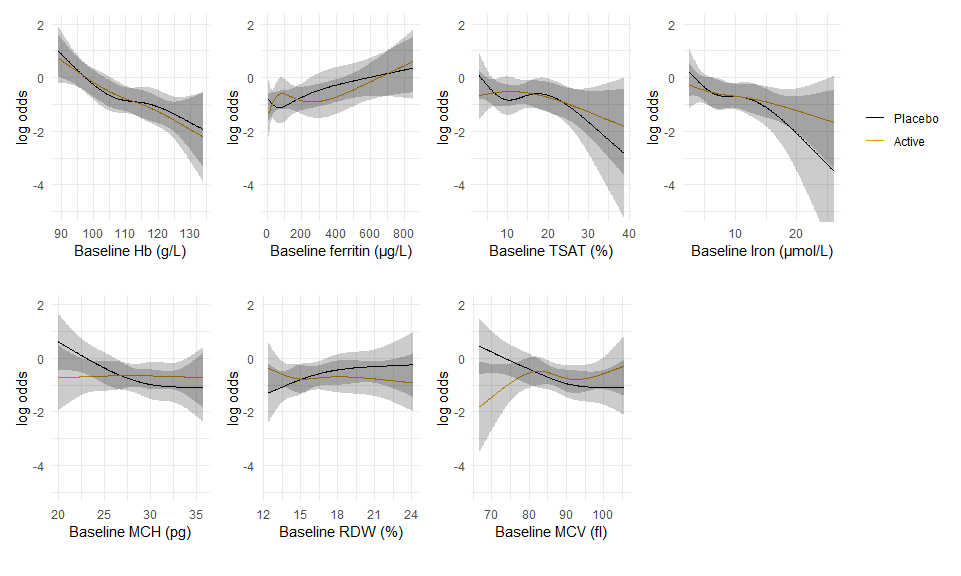


|  | **LRT P** | **Chi-square** | **df** |
| --- | --- | --- | --- |
| Baseline Hb (g/L) | 0.94 | 0.41 | 3 |
| Baseline ferritin (µg/L) | 0.44 | 2.70 | 3 |
| Baseline TSAT (%) | 0.58 | 1.99 | 3 |
| Baseline Iron (µmol/L) | 0.63 | 1.75 | 3 |
| Baseline MCH (pg) | 0.21 | 4.55 | 3 |
| Baseline RDW (%) | 0.38 | 3.08 | 3 |
| Baseline MCV (fl) | 0.07 | 7.17 | 3 |

#### Figure S2. Second primary outcome - Number of transfusions at 30 days


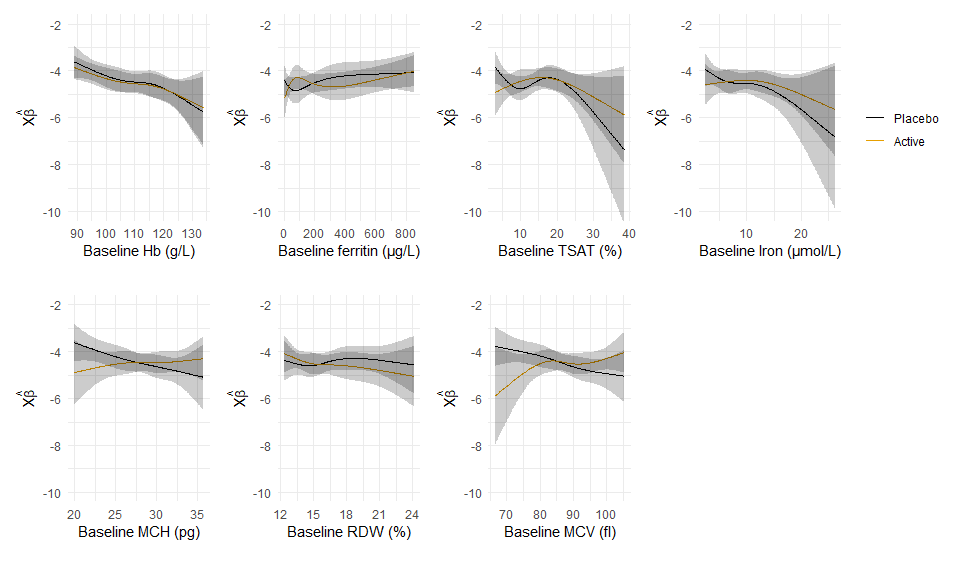


|  | **LRT P** | **Chi-square** | **df** |
| --- | --- | --- | --- |
| Baseline Hb (g/L) | 0.98 | 0.21 | 3 |
| Baseline ferritin (µg/L) | 0.24 | 4.19 | 3 |
| Baseline TSAT (%) | 0.28 | 3.84 | 3 |
| Baseline Iron (µmol/L) | 0.52 | 2.24 | 3 |
| Baseline MCH (pg) | 0.20 | 4.69 | 3 |
| Baseline RDW (%) | 0.68 | 1.49 | 3 |
| Baseline MCV (fl) | 0.07 | 7.01 | 3 |

#### Figure S3. CD grade III or above to discharge
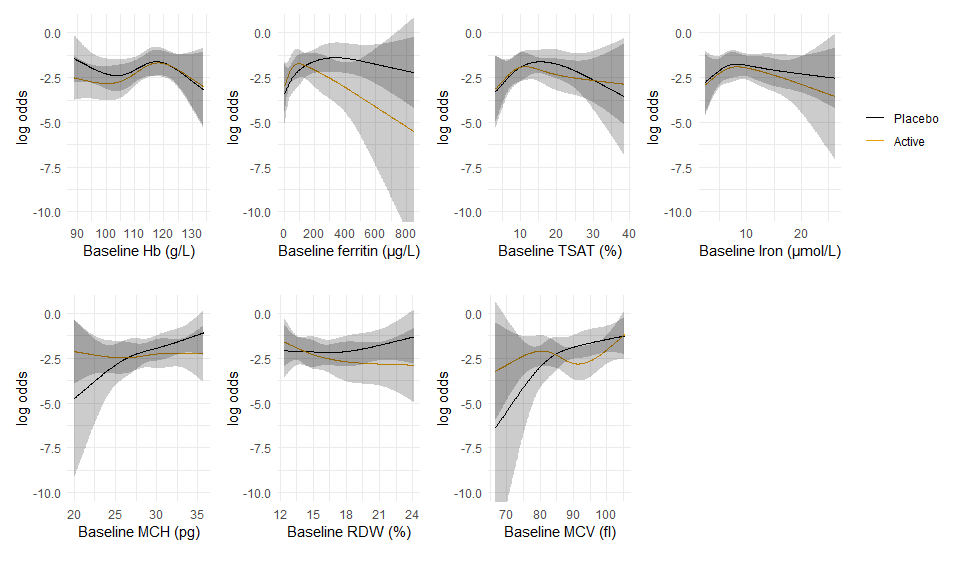


|  | **LRT P** | **Chi-square** | **df** |
| --- | --- | --- | --- |
| Baseline Hb (g/L) | 0.76 | 1.15 | 3 |
| Baseline ferritin (µg/L) | 0.18 | 4.90 | 3 |
| Baseline TSAT (%) | 0.84 | 0.82 | 3 |
| Baseline Iron (µmol/L) | 0.98 | 0.20 | 3 |
| Baseline MCH (pg) | 0.42 | 2.80 | 3 |
| Baseline RDW (%) | 0.55 | 2.10 | 3 |
| Baseline MCV (fl) | 0.23 | 4.32 | 3 |

#### Figure S4. Units of blood to 30 days (excluding LBT)


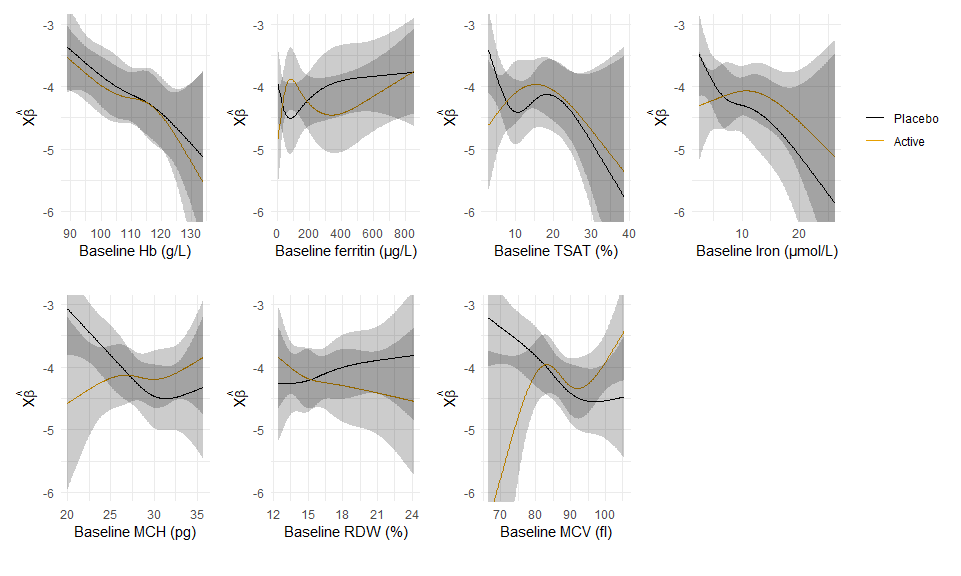


|  | **LRT P** | **Chi-square** | **df** |
| --- | --- | --- | --- |
| Baseline Hb (g/L) | 0.99 | 0.13 | 3 |
| Baseline ferritin (µg/L) | 0.13 | 5.64 | 3 |
| Baseline TSAT (%) | 0.21 | 4.54 | 3 |
| Baseline Iron (µmol/L) | 0.30 | 3.66 | 3 |
| Baseline MCH (pg) | 0.12 | 5.89 | 3 |
| Baseline RDW (%) | 0.54 | 2.15 | 3 |
| Baseline MCV (fl) | 0.03 | 8.63 | 3 |

#### Figure S5. Hospital length of stay


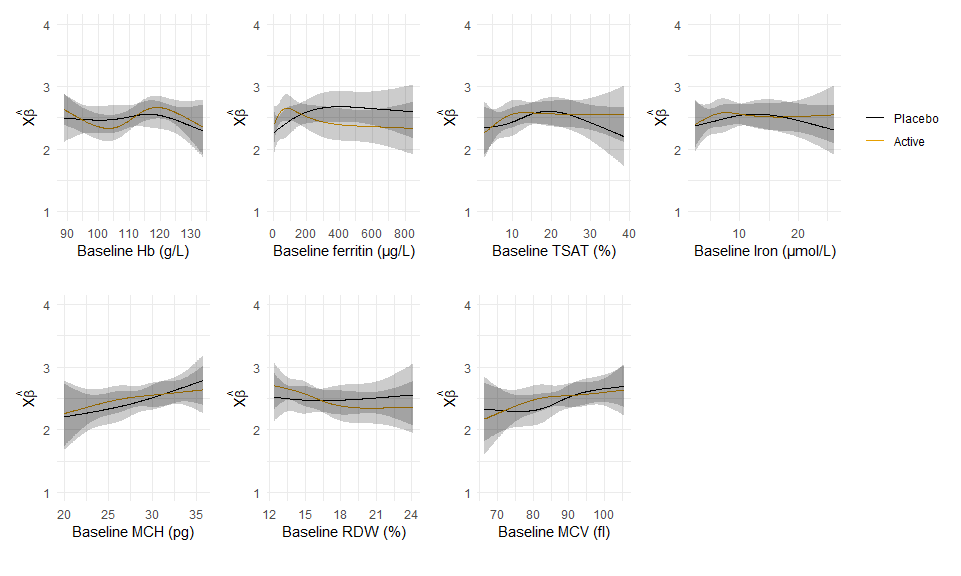


|  | **LRT P** | **Chi-square** | **df** |
| --- | --- | --- | --- |
| Baseline Hb (g/L) | 0.63 | 1.74 | 3 |
| Baseline ferritin (µg/L) | 0.14 | 5.47 | 3 |
| Baseline TSAT (%) | 0.69 | 1.49 | 3 |
| Baseline Iron (µmol/L) | 0.83 | 0.87 | 3 |
| Baseline MCH (pg) | 0.84 | 0.84 | 3 |
| Baseline RDW (%) | 0.57 | 2.03 | 3 |
| Baseline MCV (fl) | 0.75 | 1.22 | 3 |
